# The International Congress: Recent Surgical Developments

**Published:** 1913-10-25

**Authors:** 


### THE INTERNATIONAL CONGRESS.

### Recent Surgical Developments.

The recent International Congress, in drawing up the programme for its Surgical Section, was singularly fortunate in its choice of subjects for discussion. Yet it must be admitted that some of the most interesting and most informative disquisitions on modern surgical development, both in practice and in theory, and more especially, perhaps, in technique, were provided by other sections, notably that of orthopædics. This is only as it should be. General surgery is so wide and so intimately connected with sister branches that it is unavoidable that there should be some degree of overlapping. In fact it is possible that in the near future we shall see the absurdity of closely sticking to these sectional departments. All departments of medical science interlock, and sections should be concerned with treatment, diagnosis, or broad general points like these, instead of being limited to arbitrary and often impossible standards of specialty.

In a recent article in The Hospital we briefly outlined the marvellous advance which has been made during the last ten years in the surgery of the heart and greater blood-vessels. This was one of the subjects chosen for discussion at the section's first meeting, and was ably introduced by Dr. Matas, of New Orleans, who was the official "reporter" on the subject. He confined himself to that branch of the subject with which his name is most intimately connected—the cure and obliteration of aneurysms by surgical measures, chief among which is plication of the arterial wall, an operation which is now styled "Matas' aneurysmorrhaphy." More than a quarter of a century has elapsed since this operation was first successfully performed, and since then about 225 cases have been reported, in 206 of which the operation cured the aneurysm—or, rather, caused obliteration of the sac. In four cases the abdominal aorta has been attacked, but all these four cases experienced the same fatal result that attended Cooper's classical case of interference with this main blood-channel. Suture of arteries is, as we have pointed out in the article above referred to, no new method in surgery; as a matter of fact in out of the way parts of the world it is on record that farmers have successfully sutured blood-vessels in wounds inflicted on their live stock. But the Matas operation is of such importance, and its success in experienced hands is so excellent, that a discussion on its merits and a comparison between it and other and more conservative operations, such as those of Anel and Hunter, were highly desirable.

Most of the speakers agreed that the operation is advisable in certain cases in which the more conservative methods were out of the question. In the technique the great difficulty is to suture the intima of the artery with the requisite amount of rapidity and certainty that the operation demands for its success. Dr. Jeger, of Berlin, who has been working with the Trendelenburg method, exhibited his forceps, which have been designed to simplify the surgeon's work when dealing with the delicate inner lamina of the vessel. His work is particularly worthy of notice, since he has succeeded in operations on a scale of magnitude similar to that undertaken by the late Leipsic professor. Thus he has sutured the innominate artery to a branch of the pulmonary artery and successfully implanted a blood-vessel into the left ventricle of the heart. These operations have, however, all been done on animals, and they go little beyond showing the dexterity and technical skill of the operator, for it is now well established that transplantations and new junctions can be effected with success. It is only a question of technique, and the final experiment—much as some of us may cavil—must be made on patients.

Similarly interesting and instructive was the discussion on intrathoracic surgery, introduced by Professor Sauerbruch, of Zurich, who is well known as the pioneer in this department of modern surgical progress. A favourite pupil of the brilliant Breslau surgeon Mickulicz, Sauerbruch was stimulated by this master to experiment in order to find some methods whereby the collapse of the lungs, so often experienced when these viscera were surgically attacked, could be overcome. The results of his experiments were the beginnings of modern intrathoracic surgery, for the discovery of the high and low pressure operating chambers alone has made surgical interference, in the modern sense of the term, possible in severe cases of intrathoracic lesions. In his paper Professor Sauerbruch dealt exhaustively with recent work in the department of surgery of the lungs.

Among the points noted by the reporters were the usefulness of the *x*-rays in aiding the diagnosis of intrathoracic lesions; the more recent experiments with regard to pressure within the chest cavity and to absorption; the different methods and the advantages and disadvantages of high and low pressure operating; the Auer-Meltzer method of tracheal insufflation; and the recent work in regard to the surgical treatment of bronchiectasis and emphysema, which we have explained in recent articles. The discussion was illuminative. It showed that some surgeons still regard the creation of an artificial pneumothorax with grave concern, whereas it is a matter which should be considered as a small risk which, while it renders the technique more difficult, is not in itself of serious consequence to the patient. Sir William Macewen, who has had considerable experience of lung surgery, stated that in four cases he had resected a lung for phthisis; one of these patients is in excellent health eighteen years after the operation. Dr. Meltzer, of New York, criticised the Sauerbruch apparatus, and vigorously supported the method of tracheal insufflation. The latter is certainly a very valuable and useful adjunct to the surgeon, but we doubt if it is at all likely to oust the high and low pressure cabinets.

